# Myiasis Encountered in Squamous Cell Carcinoma of the Oral Cavity: A Case Report

**DOI:** 10.7759/cureus.62414

**Published:** 2024-06-15

**Authors:** Hassam Zaka Khan, Syeda Aliza Shahid, Nausheen Bakht, Syed Shahid Nafees Zaidi

**Affiliations:** 1 Anatomy, Combined Military Hospital (CMH) Lahore Medical College and Institute of Dentistry, Lahore, PAK; 2 Pathology, Combined Military Hospital (CMH) Lahore Medical College and Institute of Dentistry, Lahore, PAK; 3 Epidemiology and Public Health, Armed Forces Postgraduate Medical Institute, Rawalpindi, PAK; 4 Cardiac Surgery, Rawal Institute of Health Sciences, Islamabad, PAK

**Keywords:** myiasis infestation, wound infections, oral cavity squamous cell carcinoma, wound care management, oral myiasis

## Abstract

Myiasis is a rare parasitic condition, caused by fly larvae infesting human tissues. Its consequences can be severe, as deafness, blindness, extensive tissue loss, and even death can occur due to the infestation.

We present a case of myiasis in a 62-year-old Pakistani woman with advanced well-differentiated oral squamous cell carcinoma (OSCC) undergoing palliative chemotherapy. The patient presented with an extensive, necrotic lesion in the submental and submandibular region infested with live larvae. Management included mechanical removal over three sessions and ivermectin. Once all the larvae were eradicated, the patient was referred to the plastic surgery department for reconstruction.

This case report highlights the importance of maintaining a high index of suspicion for myiasis in patients with OSCC, particularly those with extensive ulcerated lesions. Educating at-risk individuals and healthcare providers on myiasis and the importance of wound hygiene is crucial for reducing the burden of this preventable complication.

## Introduction

Myiasis refers to the contamination of human tissue with fly larvae. It is a rare condition predominantly seen in tropical and subtropical areas [1]. Risk factors for developing myiasis include poor hygiene, poor sanitation, malnutrition, and low socioeconomic status as well as comorbidities like malignant wounds, diabetes, peripheral vascular disease, and psychiatric illnesses [2]. 

Oral squamous cell carcinoma (OSCC) results in various morbidities such as pain, bleeding on provocation, loss of function, facial disfigurement, extra-oral fungation, and tissue necrosis [3]. In susceptible individuals, this necrotic tissue attracts flies to lay their eggs [4]. 

Wound myiasis presents with symptoms such as pain, drainage, odor, edema, bleeding, and psychosocial problems [5]. When this infestation occurs in the head and neck region, it can have disastrous consequences, including widespread tissue destruction, blindness, deafness, and even death [6]. Therefore, it is important to recognize the issue and address it promptly.

This case report aims to outline a case of myiasis associated with squamous cell carcinoma in a 62-year-old Pakistani woman and discuss the adequate treatment for it.

## Case presentation

A 62-year-old woman presented to the ENT clinic of our hospital, with a history of a non-healing ulcer on the floor of mouth for the past six months. 

On external examination, there was a hard, fixed, and non-tender swelling approximately 6 x 4 cm in size, involving the bilateral submental and the submandibular region. On examination of the oral cavity, there was an ulcerative region on the left lower alveolus. It was approximately 4 x 2 cm in size, extending from the first to the last molar, sparing the retro-molar trigone. The rest of the floor of mouth was normal and mouth opening was adequate. A biopsy was planned which resulted in a diagnosis of well-differentiated squamous cell carcinoma of the left lower alveolus. There were no other comorbidities. She was subsequently referred to the oncology department of our hospital for palliative chemotherapy which was initiated with paclitaxel and carboplatin in April 2023 with 21-day cycles.

**Figure 1:**
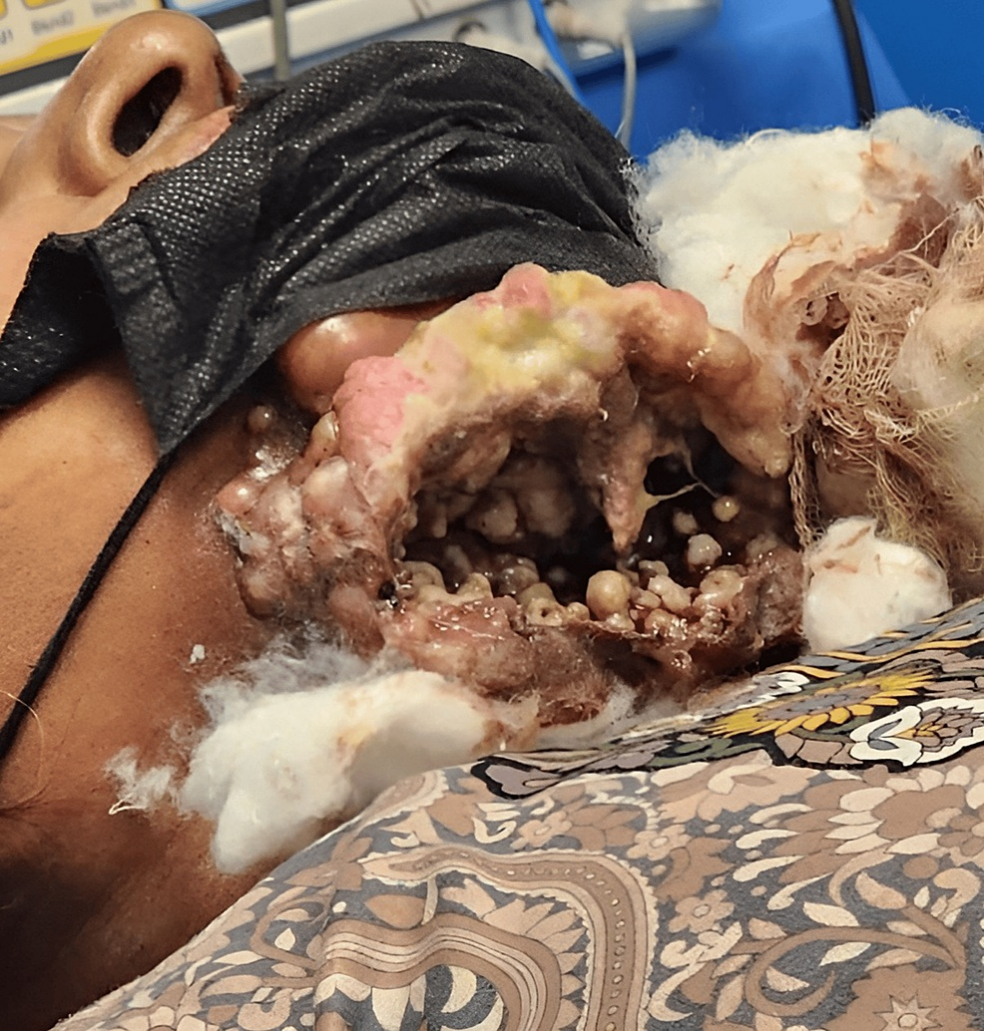
Extensive soft tissue defect involving the submental and submandibular regions

According to the patient, after receiving the third dose of chemotherapy, a discharging sinus developed between the ulcer and her neck. The emergency department dressed the wound and urgently directed her to follow up at our hospital's ENT clinic. However, she presented to the clinic, three weeks later, with an extensive lesion involving the submental and submandibular regions of the neck. The lesion was necrotic with elevated edges, there was a loss of overlying skin substance, and there were live larvae inside the lesion (Figure 1). 

The patient admitted to self-dressing her wounds for three weeks, avoiding medical attention due to embarrassment. The malodor associated with the patient's advanced maggot infestation ultimately resulted in the patient’s family taking her to the hospital.

Initially, turpentine-soaked dressings were applied to the wound. This was followed by the manual removal of larvae with forceps (Video 1). After removal, the wound was washed with saline and dressed with 10% povidone-iodine. The larvae were removed on every alternate day, in three sessions. The patient was sent home after each session, with instructions about wound care. She was also instructed to keep her house free of flies. The patient was given 6 mg ivermectin, once daily for three days along with 400mg Ibuprofen on an as-needed basis. No antibiotics were initiated. The number of live larvae was reduced after the first visit and on the third follow-up, only dead maggots remained. On each follow-up visit, the degree of odor and exudation from the wound was less than the preceding one. Following her last visit, when it was ensured that all larvae were removed, she was referred to the plastic surgery department for consultation regarding reconstruction as the infestation had caused enormous tissue loss. Unfortunately, the patient's prognosis is unknown as she did not come for a follow-up after the four initial treatment sessions.

**Video 1:** Larval removal

## Discussion

Myiasis is derived from the Greek word 'myia' meaning 'fly' and refers to fly larvae invading vital human or other mammalian tissues [7]. Various local and systemic factors predispose to the condition. The local factors include poor oral hygiene, incompetent lips, periodontal disease, and halitosis [7] while the systemic issues include neurological deficits, psychiatric illnesses, alcoholism, diabetes, and malignancy [2]. There is also a social element to the disease as most patients have a low socioeconomic and educational background. Many of them are elderly and often victims of neglect and social isolation [3]. 

In cases of myiasis associated with underlying malignancy, most patients present with advanced and neglected tumors [2,8]. Our patient was also a case of locally advanced OSCC. Malignant wounds create a suitable environment for larvae to develop. Their necrotic debris attracts flies which lay their eggs in the wound. These eggs subsequently penetrate deeper tissues and develop into larvae [4]. In addition to the malignancy itself, chemotherapy predisposes to myiasis by creating an immunocompromised state in the host and can worsen underlying malnutrition [9]. 

Our patient presented to our clinic in June 2023, in Lahore Pakistan, when temperatures averaged 40°C. This aligns with the consensus that myiasis is more common in hot and humid climates [4]. Cases of myiasis typically involve patients from rural areas with limited access to healthcare [10,11]. However, our patient possessed health insurance that entitled her to free treatment at our tertiary care hospital. Despite having health insurance, she failed to seek timely medical attention or maintain proper wound hygiene. Furthermore, the patient's secrecy around her condition and reliance on her family for seeking medical attention illustrates how social isolation can increase the risk of myiasis. It also highlights the importance of strong support systems in facilitating timely access to healthcare when patients may struggle to do so themselves.

The treatment of myiasis includes mechanical removal of the larvae, surgical debridement, antibiotics, and anti-helminthic drugs [3,11]. Tetanus prophylaxis has also been recommended [12]. However, the mainstay of treatment is the mechanical removal of larvae and other modalities can be added based on individual patient assessment [3]. Sometimes mechanical removal is insufficient in removing all maggots and asphyxiants such as turpentine oil can be used to drive larvae out of the tissue. Other agents, such as mineral oil, ether, chloroform, ethyl chloride, mercuric chloride, creosote, saline, phenol, calomel, olive oil, and iodoform, have also been recommended for this purpose [13]. 

Ivermectin, an anti-helminthic drug, has been used against many parasitic diseases including myiasis. Its anti-parasitic action results from creating a prolonged period of hyperpolarization which decreases the number of action potentials in the parasite [14]. This paralyzes the larvae, which can then be removed manually. All larvae may not be removed in one session, so frequent follow-up is important [13]. Our patient was maggot-free on her fourth follow-up visit. 

As a consequence of both the malignancy and myiasis, our patient was left with a large disfiguring wound and she was referred to the plastic surgery department of our hospital for possible reconstruction. Apart from aesthetically displeasing wounds, several other consequences of the condition have been reported in the literature, including mortality, tooth loss, bone defects, maxillectomy, hearing impairment, vision loss, oroantral communication, irreversible injury to the salivary glands, and paresthesia [15]. After the initial treatment sessions and referral to plastic surgery, the patient did not return for follow-up. Therefore, we cannot comment on the patient's wound healing and overall prognosis. Recognizing this limitation, we recommend that healthcare professionals dealing with wound myiasis patients establish a comprehensive follow-up plan, extending beyond achieving initial larval clearance. This recommendation is important because these patients may neglect to seek further medical attention.

Prompt diagnosis and proper wound management are crucial to prevent myiasis and its complications in patients with extensive OSCC. In addition to proper wound care, sanitation measures play a significant role in preventing fly infestation. These measures include using mosquito nets to create a physical barrier for flies, limiting their entry to the wound site and keeping the surroundings clean and free of garbage. Implementing these preventive measures can reduce the risk of myiasis in vulnerable patients.

## Conclusions

Our case report highlights myiasis as a potential complication of OSCC, especially in individuals with large, open wounds. While traditionally seen in resource-limited settings, our case emphasizes the need for vigilance in all healthcare environments. Healthcare professionals should educate patients with OSCC and their families to recognize alarming symptoms such as foul odor, bleeding, or increased pain and seek timely medical attention. Proper wound dressing and patient education on wound care and sanitation measures are crucial to prevent complications. 
